# Vaginal douching and racial/ethnic disparities in phthalates exposures among reproductive-aged women: National Health and Nutrition Examination Survey 2001–2004

**DOI:** 10.1186/s12940-015-0043-6

**Published:** 2015-07-15

**Authors:** Francesca Branch, Tracey J. Woodruff, Susanna D. Mitro, Ami R. Zota

**Affiliations:** Department of Environmental and Occupational Health, Milken Institute School of Public Health, George Washington University, 950 New Hampshire Avenue NW, Suite 414, Washington DC, 20052 USA; Program on Reproductive Health and the Environment, Department of Obstetrics, Gynecology, and Reproductive Sciences, University of California, San Francisco, Oakland, CA USA

**Keywords:** Biomonitoring, Diethyl phthalate, Endocrine disruptors, Environmental justice, Feminine hygiene, Fragrance, Health disparities, NHANES, Personal care products, Phthalates

## Abstract

**Background:**

Diethyl phthalate (DEP) and di-*n*-butyl phthalate (DnBP) are industrial chemicals found in consumer products that may increase risk of adverse health effects. Although use of personal care/beauty products is known to contribute to phthalate exposure, no prior study has examined feminine hygiene products as a potential phthalate source. In this study, we evaluate whether vaginal douching and other feminine hygiene products increase exposure to phthalates among US reproductive-aged women.

**Methods:**

We conducted a cross-sectional study on 739 women (aged 20–49) from the National Health and Nutrition Examination Survey 2001–2004 to examine the association between self-reported use of feminine hygiene products (tampons, sanitary napkins, vaginal douches, feminine spray, feminine powder, and feminine wipes/towelettes) with urinary concentrations of monoethyl phthalate (MEP) and mono-n-butyl phthalate (MnBP), metabolites of DEP and DnBP, respectively.

**Results:**

A greater proportion of black women than white and Mexican American women reported use of vaginal douches, feminine spray, feminine powder, and wipes/towelettes in the past month whereas white women were more likely than other racial/ethnic groups to report use of tampons (p < 0.05). Douching in the past month was associated with higher concentrations of MEP but not MnBP. No other feminine hygiene product was significantly associated with either MEP or MnBP. We observed a dose–response relationship between douching frequency and MEP concentrations (p_trend_ < 0.0001); frequent users (≥2 times/month) had 152.2 % (95 % confidence intervals (CI): (68.2 %, 278.3 %)) higher MEP concentrations than non-users. We also examined whether vaginal douching mediates the relationship between race/ethnicity and phthalates exposures. Black women had 48.4 % (95 % CI: 16.8 %, 88.6 %; p = 0.0002) higher MEP levels than white women. Adjustment for douching attenuated this difference to 26.4 % (95 % CI:−0.9 %, 61.2 %; p = 0.06). Mediation effects of douching were statistically significant for black-white differences (z = 3.71, p < 0.001) but not for differences between Mexican Americans and whites (z = 1.80, p = 0.07).

**Conclusions:**

Vaginal douching may increase exposure to DEP and contribute to racial/ethnic disparities in DEP exposure. The presence of environmental chemicals in vaginal douches warrants further examination.

## Background

Phthalate acid esters, also known as phthalates, are a class of multifunctional industrial chemicals that warrant public health concern due to their ubiquitous presence in the environment and their potential effects on human health. Low molecular weight phthalates such as diethyl phthalate (DEP) and di-*n*-butyl phthalate (DnBP) are used in fragranced personal care products such as cologne/perfume, hair products, deodorant, lotion, body wash, and nail polish to retain scents [1, 2]. Because phthalates are not chemically bound to products, product use can lead to phthalates exposure through dermal absorption, inhalation, and ingestion. Once in the body, phthalates are quickly metabolized and excreted in urine, with elimination half-lives less than 24 h [3, 4]. Urinary metabolites of DEP and DnBP are detected in 99 % of US women of reproductive age [5].

Exposure to DnBP and DEP is associated with adverse effects on reproductive, endocrine, and developmental systems. Experimental animal studies find that DnBP, but not DEP, has well-documented anti-androgenic effects on the reproductive systems of male offspring exposed in utero [6]. The female reproductive system is thought to be less sensitive to phthalate-induced disruption than the male reproductive system. However a recent study that examined the effects of a phthalate mixture (which included DnBP but not DEP) on female rat offspring exposed in utero reported reproductive malformations consistent with the Mayer–Rokitansky–Kuster–Hauser syndrome [7]. Moreover, a recent study of DEP toxicity found evidence of estrogenicity in both in vitro and in vivo, in adult female rats, suggesting that DEP may induce reproductive abnormalities in female reproductive system by both genomic and non-genomic mode of actions [8]. The evidence of health effects in humans is still evolving; however, epidemiologic studies suggest that prenatal DnBP and DEP exposure is associated with sex steroid hormone concentrations during pregnancy [9] and may increase the risk of adverse health outcomes during early childhood [10-12]. In adult women, DnBP exposure is associated with increased risks of diabetes [13] and endometriosis [14] while DEP exposure is associated with increased biomarkers of oxidative stress [15], elevated mammographic breast density and increased breast cancer risk [16].

Phthalates exposure varies by race/ethnicity and socioeconomic status (SES). Black females and males have higher levels of DEP metabolites than their white counterparts [17-20] and lower SES is associated with elevated levels of DnBP [17, 21, 22] though reasons underlying social disparities in phthalates exposures remain poorly understood. Mounting evidence points to environmental factors in shaping racial/ethnic and socioeconomic disparities in reproductive health, so these disparities in phthalates exposure may contribute to disparities in health outcomes [23].

Given the concern over phthalate toxicity, it is important to identify modifiable sources of phthalates exposure that may be targeted for exposure reduction strategies, particularly for vulnerable populations. Personal care product use is considered an important exposure pathway for DEP and DnBP [24], particularly among reproductive-aged women who use multiple products daily [25]. Numerous studies [10, 19, 25-27] report an association between use of colognes/perfumes and other fragranced products and higher urinary concentrations of mono-ethyl phthalate (MEP), a metabolite of DEP, with MEP concentrations in fragrance users approximately 3 times that of non-users [25]. Associations between personal care products and MnBP, the primary metabolite of DnBP, are less consistent. Among the studies that examined these relationships, five found a positive association between some type of personal care product use and urinary MnBP [10, 25, 27-29], three reported no association [25, 26, 28], and one found an inverse association [19].

Feminine care products that are used vaginally, such as douches, tampons, and deodorants, may be an unrecognized exposure source of phthalates. In particular, vaginal douching, the practice of intravaginal cleansing with a liquid solution, warrants consideration since most commercial douches contain fragrance [30] and synthetic fragrances are a mixture of compounds that often contain phthalates [1, 31, 32]. The practice of vaginal douching may also play a role in shaping chemical exposure disparities, since black women report douching more frequently than white and Mexican American women [18, 19].

Although socio-cultural differences in personal care product use have been hypothesized as a possible driver of disparities in phthalates exposure [21], to our knowledge, no study to date has characterized this relationship. We conducted a cross-sectional study to examine the association between self-reported use of vaginal douches and other feminine hygiene products with DEP and DnBP metabolites, MEP and MnBP respectively, among a nationally representative sample of U.S. reproductive aged women. We also examined whether differences in douching would partially explain racial/ethnic differences in phthalate metabolites levels.

## Methods

### Study population

We pooled data from the 2001–2002 and 2003–2004 cycles of the National Health and Nutrition Examination Survey (NHANES), a nationally representative survey and physical examination of the civilian, non-institutionalized U.S. population conducted by the US Centers for Disease Control and Prevention (CDC). These cycles were the only ones in which data were collected on use of feminine hygiene products. The sample was restricted to female participants aged 20–49 who had self-reported data on feminine hygiene product use (n = 2432). Of these, 805 participants had urinary measurements of MEP and MnBP. We excluded participants who did not self-identify as non-Hispanic white, non-Hispanic black, or Mexican American (n = 66) resulting in a final sample size of 739 study participants.

### Phthalate metabolite measurements

Phthalate metabolites were measured in approximately one-third of NHANES participants. Concentration data for MEP and MnBP were downloaded from the NHANES website in September 2013. Spot urine samples were collected in the Mobile Examination Center and stored at −20 °C until shipped to CDC’s National Center for Environmental Health for analysis. Analytical methods have been described in detail elsewhere [33, 34]. Briefly, concentrations of phthalate metabolites were quantified using solid phase extraction-high performance liquid chromatography-isotope dilution-tandem mass spectrometry and expressed as wet weights (ng/mL). Approximately 99 % of the urinary phthalate metabolite data was above the limit of detection (LOD); values below the LOD were substituted with LOD/by the CDC [33, 34].

Feminine hygiene product use data. As part of the 2001–2004 NHANES survey, reproductive-aged females were asked the following question during a private face-to-face interview, “During the past month, have you used any of the following products for feminine hygiene: tampons, sanitary napkins, vaginal douches, feminine spray, feminine powder, feminine wipes/towelettes, or other feminine hygiene products?” If the participant responded “yes”, she was then asked about the use of each specific product. From these questions, we created binary variables (yes/no) for each type of product used (e.g. tampons, vaginal douches, etc.)

Data on frequency of use was available for vaginal douche use (but not any other feminine hygiene products). Douching frequency was assessed from the following survey questions: “During the past 6 months, did you douche? By douching we mean putting a substance into your vagina either for routine cleansing or for vaginal irritation or signs of infection?” If participants responded yes, they were then asked, “During the past 6 months, how often did you douche?” We used this data to classify participants into one of three categories indicating intensity of use: no use, occasional use (douche ≤ once per month), or frequent use (douche ≥2 times per month).

### Statistical analysis

Analyses were conducted in STATA v13.1. Because we combined two survey cycles, we calculated new sample weights for each participant equal to one half of the two-year sample weights provided in the NHANES laboratory files. All analyses were adjusted for the non-random sampling design and the sample population weights. A (two-sided) P-value <0.05 was considered statistically significant.

We tested our primary hypothesis using multivariate linear regression models where the outcome was individual phthalate metabolites and the main exposure variable was use of individual feminine hygiene products. We natural log-transformed phthalate metabolite and creatinine data prior to regression analysis to account for their non-normal distributions. From these regression models, we estimated the percent changes in phthalate metabolite concentrations by product use as (e^(β)^ −1) * 100 with 95 % confidence intervals (CIs) estimated as (e^(β ± 1.99 * SE)^ −1) where β and SE are the estimated regression coefficient and standard error, respectively.

We first examined multivariate associations between phthalate metabolite concentrations and feminine hygiene product use adjusted only for urinary creatinine concentrations (to account for urinary dilution) [35]. We then assessed associations accounting for the following covariates and potential confounders: age (continuous); body mass index (BMI; continuous); race/ethnicity (non-Hispanic white, non-Hispanic black, and Mexican American); and educational attainment (less than high school diploma; high school diploma or equivalent; or more than high school diploma). We also conducted a sensitivity analysis by further adjusting for household poverty-income ratio (PIR; the ratio of household income to poverty threshold adjusted to family size and inflation) among the 698 participants with available data.

Lastly, we evaluated whether douche use partially mediated the association between race/ethnicity and phthalate exposure in two ways. We first qualitatively examined whether the effect estimate of race/ethnicity on phthalate metabolite concentrations changed by greater than ten percent after controlling for douching frequency. We then formally tested for mediation using the Arioan test for mediation using methodological extensions to accommodate categorical mediators [36, 37].

## Results

Phthalate metabolite concentrations, feminine hygiene product use, and demographic characteristics varied by race/ethnicity among US reproductive-aged women (Table 1). Black women were more likely to be obese, and Mexican American women had the lowest educational attainment. Compared to other racial/ethnic groups, white women self-reported significantly higher use of tampons, whereas black women self-reported significantly higher use of the following feminine hygiene products: vaginal douche, feminine spray, feminine powder, wipes/towelettes, and other products (p < 0.05). For example, 37 % of black women reported douching in the previous month compared to 14 and 10 % of white and Mexican-American women, respectively (Table 1). Similarly, black women reported douching more frequently than other women (p < 0.001). Twenty percent of black women reported douching frequently compared to 7 and 3 % in white and Mexican-American women, respectively. Black women also had significantly higher geometric mean concentrations (ng/mL) of MEP and MnBP when compared to white or Mexican-American women (p <0.001).

**Table 1:** Demographic characteristics, product use, and phthalate metabolite concentrations by race/ethnicity among US reproductive-aged women, NHANES 2001–2004 (N = 739)

	White (N = 396)	Black (N = 163)	Mexican American (N = 180)	*p*-value^a^
Age (years), %				
20-29	29	34	42	0.05
30-39	33	32	32	
40-49	38	34	26	
BMI (kg/m2), %^b^				<0.001
<25	44	24	26	
25 – 30	27	25	33	
≥30	29	52	41	
Educational attainment, %				<0.001
< HS graduate	9	25	47	
HS graduate	22	24	21	
> HS graduate	69	52	31	
Feminine hygiene product use in past month^c^, %				
Tampon	55	31	22	<0.001
Sanitary Napkin	59	66	63	0.13
Vaginal Douche	14	37	10	<0.001
Feminine Spray	3	16	2	<0.001
Feminine Powder	2	12	2	<0.001
Wipes/Towelettes	7	16	13	0.004
Other Products	3	11	5	0.03
Average douching frequency in past 6 months, %				<0.001
None	83	47	86	
Occasional (≤1 / month)	11	33	11	
Frequent (≥2/month)	7	20	3	
Phthalate metabolite concentration (GM (GSE))^d^				
Mono-ethyl phthalate (MEP; ng/mL)	127 (10.7)	268 (26.5)	247 (26.1)	<0.001
Mono-butyl phthalate (MnBP; ng/mL)	18.2 (1.0)	32.3 (2.0)	23.7 (2.3)	<0.001

^a^Group differences evaluated by ANOVA for natural log transformed phthalate metabolite concentrations and chi square test for categorical variables

^b^Data missing for BMI (n =8)

^c^Some participants used multiple products

^d^ *GM*, Geometric mean; *GSE*, Geometric standard error

Douching was associated with increased urinary MEP concentrations (Table 2) in both unadjusted and adjusted models. For example, in adjusted models, women that reported douching in the past month had 51.6 % (95 % CI: 18.8 %, 93.6 %) higher concentrations of MEP than non-users. We also observed evidence of a monotonic dose response relationship between douching frequency and MEP concentrations (p-trend <0.001). In adjusted models, women who reported occasional douche use in the past 6 months had 33.6 % (95 % CI: −0.3 %, 79.2 %) higher MEP concentrations than non-users, and those who reported frequent douche use had 152.2 % (95 % CI: 68.2 %, 278.3 %) higher MEP concentrations than non-users. There was evidence of a positive association between douching and MnBP but it did not reach statistical significance. There were no other significant associations between the other feminine hygiene products and either MEP or MnBP although effect estimates were large for feminine spray, feminine powder, and other products. Associations did not substantially change when we adjusted for household income (data not shown).

**Table 2:** Associations of product use and phthalate metabolite concentrations (ng/mL) among US reproductive-aged women, NHANES 2001-2004^a^

	MEP				MnBP			
	Unadjusted (N = 739)		Adjusted (N = 731)		Unadjusted (N = 739)		Adjusted (N = 731)	
	% change	95 % CI	% change	95 % CI	% change	95 % CI	% change	95 % CI
Feminine hygiene product use in past month								
Tampon^b^	−6.4	(−24.9, 16.6)	6.1	(−16.0, 35.5)	2.4	(−11.8, 18.9)	4.1	(−11.0, 21.9)
Sanitary napkin^b^	−7.6	(−21.5, 8.7)	−9.0	(−22.8, 7.2)	−3.2	(−17.6, 13.6)	−4.4	(−18.9, 12.7)
Vaginal douche^b^	59.5^*^	(22.7, 107.2)	51.6^*^	(18.8, 93.6)	12.2	(−8.3, 37.3)	9.8	(−9.7, 33.6)
Feminine spray^b^	79.7	(−2.6, 231.4)	63.2	(−12.4, 204.2)	26.4	(−15.0, 88.1)	20.7	(−21.0, 84.3)
Feminine powder^b^	64.9	(−16.4, 225.3)	49.4	(−23.9, 193.2)	23.7	(−25.7, 105.9)	16.6	(−32.1, 100.3)
Wipes/ Towelettes^b^	21.5	(−11.1, 65.9)	14.7	(−16.0, 56.7)	−6.5	(−25.8, 17.9)	−7.8	(−27.9, 17.9)
Other products^b^	35.4	(−12.2, 108.7)	26.9	(−19.8, 100.8)	27.8	(−7.5, 76.7)	25.9	(−7.9, 72.1)
Average douching frequency in past 6 months^b^								
Occasional (≤1 month)	40.5^*^	(6.1, 86.1)	33.6	(−0.3,79.2)	7.7	(−5.6, 22.8)	8.2	(−4.5, 22.6)
Frequent (≥2 month)	154.4^**^	(68.7, 283.6)	152.2^**^	(68.2, 278.3)	29.0	(−6.2, 77.3)	24.1	(−10.3, 71.8)
P-trend		<0.001		<0.001		0.094		0.145

^a^MEP and MnBP concentrations were natural log transformed. All models were adjusted for urinary creatinine; adjusted models additionally controlled for age, race/ethnicity, BMI, and educational attainment

^b^Reference group is non-users; ^*^p < 0.05; ^**^ p < 0.001

To further understand whether douching partially explains the relationship between race/ethnicity and phthalate metabolite levels, we modeled two linear regression models for MEP, with and without douching frequency as a covariate (Table 3). In the covariate-adjusted model that did not account for douching, black women had 48.4 % higher concentrations of MEP (95 % CI: 16.8 %, 88.6 %), and Mexican American women had 58.2 % higher MEP concentrations (95 % CI: 24.7 %, 100.8 %) compared to white women. After controlling for douching frequency, the effect estimate for black women decreased to 26.4 % (95 % CI: −0.9 %, 61.2 %), and was no longer statistically significant, while the effect estimate for Mexican American women increased to 70.1 % (95 % CI: 34.1 %, 115.9 %). Mediation effects of douching were statistically significant for black-white differences (z = 3.71, p < 0.001) but not statistically significant for differences between Mexican Americans and whites (z = 1.80, p = 0.07).

**Table 3:** Association between race/ethnicity and MEP (ng/mL) among US reproductive-aged women with and without adjustment for douching frequency, NHANES 2001-2004

	Model 1 (N = 731)		Model 2 (N = 731)	
	% change	95 % CI	% change	95 % CI
Race/Ethnicity^a^				
Black	48.4^*^	(16.8, 88.6)	26.4	(−0.9, 61.2)
Mexican-American	58.2^**^	(24.7, 100.8)	70.1^*^	(34.1, 115.9)
Age	−0.1	(−1.2, 1.0)	−0.4	(−1.5, 0.7)
Educational attainment^b^				
< HS graduate	19.0	(−20.2, 77.5)	6.6	(−26.7, 54.9)
HS graduate	30.7^*^	(3.4, 65.1)	27.7^*^	(2.3, 59.3)
BMI	0.3	(−0.9, 1.5)	0.2	(−1.0, 1.4)
Urinary creatinine	129.4^**^	(92.3, 173.8)	124.5^**^	(88.2, 167.9)
Douching frequency in past six months^c^				
Occasional (≤1 month)	-	-	33.6	(−0.3, 79.2)
Frequent (≥2 month)	-	-	152.2^**^	(68.2, 278.3)
R^2^	0.24		0.27	

^a^Reference group is white women

^b^Reference group is > HS graduate

^c^Reference group is non douche users; ^*^ *p* < 0.05; ^**^ *p* < 0.001

## Discussion

In this cross-sectional study of US reproductive aged-women, we found that vaginal douching may be an important source of DEP exposure among US reproductive aged women. In adjusted models, women who reported douching in the past month had 52 % higher urinary MEP concentrations than non-users. In addition, there was evidence of a monotonic dose–response relationship such that women who reported an average douching frequency of two or more times a month had urinary MEP concentrations that were 152 % higher than nonusers.

We found no associations between MEP and the other feminine hygiene products. There are many possible reasons for the lack of associations found. For example, scented products used intravaginally may result in greater chemical exposures than scented feminine hygiene products designed for external use. Alternatively, it is possible that, despite the large point estimates (see Table 2), the wide confidence intervals for the other feminine hygiene products could reflect the larger variety of scented and unscented products in these different categories relative to those available for douching; unfortunately, specific brand information and detail about scent was not collected in NHANES.

We found no association between douching, or any other product use, and urinary MnBP concentrations. This is consistent with most epidemiologic studies that report stronger associations between fragranced personal care products and MEP than MnBP. This may in part be explained by the fact that, on average, higher concentrations of DEP than DnBP are present in personal care products [1, 2].

We observed racial/ethnic differences in phthalate exposures as well as feminine hygiene practices consistent with prior studies [21, 26]. Compared to white and Mexican American women, we found that black women had higher phthalate metabolite levels and were more likely to report use of several feminine hygiene products including douches, sprays, and powders. When we controlled for frequency of douching, differences in urinary MEP concentrations between black and white women were attenuated and no longer statistically significant, suggesting that use of personal care products, such as vaginal douches, may contribute to racial/ethnic disparities in phthalates exposures. In contrast, Mexican American women had higher MEP concentrations than white women, but these differences were not explained by douching, indicating the importance of other exposure sources among Mexican Americans.

The reasons underlying racial/ethnic differences in douching behavior are complex and likely involve individual as well as community-level drivers. Among African American and white women, common motivations for douching include: to feel fresh and clean, to remove menstrual blood, to remove vaginal odors, after sexual activity, and in response to vaginal discharge or irritation [30, 38]. Ferranti hypothesizes that structural forces, such as historical odor discrimination against African Americans and targeted advertising of these products to African Americans, have helped to establish the practice of douching as a beauty norm specifically among the African American community, and that norm is now perpetuated through cultural and familial traditions [39]. Thus, environmental justice advocates and practitioners may view the disproportionate chemical exposures from douching as an “environmental injustice of beauty*”*, a framework that could also be applied to other beauty practices used predominately by women of color such as hair straightening and skin whitening.

Vaginal douching is associated with multiple adverse health outcomes including increased risks of bacterial vaginosis [40], pelvic inflammatory disease [41], and possibly cervical cancer [42], and consequently this practice has been discouraged by the American College of Obstetrics and Gynecology [43,44]. While these guidance documents do not discuss potential chemical hazards and their consequent health impacts on reproductive health, recognition of this and other types of environmental risk factors is growing among health care providers, and was recently addressed in an ACOG Committee Opinion entitled “Exposure to Toxic Environmental Agents” [45].

As our study did not make any direct connection to adverse health consequences, the human health implications of our findings warrant further research. While experimental animal studies suggest that DEP is not a male reproductive toxicant [46], several epidemiologic studies have reported associations between maternal concentrations of MEP during pregnancy and adverse reproductive and developmental outcomes in their offspring, particularly males [10, 11, 47]. A recent epidemiologic study of adolescent girls found suggestive associations between both in utero and peripubertal MEP concentrations and earlier pubertal onset [48]. The disparity between the results in rodent and human studies has not been explained, but may reflect differences in exposure route (oral administration in animals versus dermal or inhalation exposure in humans), or may be due to other biological differences between rodents and humans [25]. Moreover, localized effects of DEP (or other phthalates) on the vaginal epithelium or the vaginal microbiome have not been fully considered in prior toxicological or epidemiologic studies. Given the high levels of MEP and the widespread exposure in US reproductive-aged women, its effect on vaginal health warrant further consideration.

Our current study focused solely on phthalates exposure; however, the vaginal route of exposure may also be important for other chemicals found in these products. Fragrance is typically a mixture of chemicals [49]. DEP was a common component of fragrance, but a recent study suggests that DEP exposure is declining in the US population [50] and thus manufacturers may be replacing it with other chemicals. In addition, preservatives such as parabens and antimicrobial agents such as triclosan, which have also been shown to have endocrine-disrupting properties, may be present in intravaginal products [51]. Further research is needed to understand the effects of chemical exposures on reproductive health since little is known about the absorption and metabolism of environmental chemicals that are vaginally administered. A small pharmaceutical study found that serum estradiol concentrations were ten times higher when the same dose was delivered via vaginal administration compared to oral administration [52]. The differential uptake observed for synthetic hormones may also apply to industrial chemicals since many of them are suspected endocrine disrupters. Further work should also examine how variations in vaginal microbiota may change metabolism and absorption of environmental chemicals since douching can alter the chemical and biological environment of the vagina [53].

The cross-sectional nature of the study precludes the ability to make any causal inferences about the direction of the association between urinary MEP concentration and douching. Additionally, there may be residual confounding from unaccounted phthalate sources. Higher MEP levels have been associated with use of multiple personal care products such as perfumes/colognes, cosmetics, and hair care products [19, 25, 27]. Data on these potential sources of exposures were not available in NHANES. It is possible that women who frequently douche are also more likely to use other scented personal care products. Among our study population, women who douched reported using more feminine hygiene products than those who did not douche (data not shown). However, the observed dose–response relationship coupled with the null associations with the other feminine hygiene products supports a plausible relationship that warrants investigation in future studies. Another limitation of this study is that we did not have information on the chemical ingredients of the douches used by study participants. For example, homemade douching solutions comprised of vinegar and water would be less likely to have phthalates than commercially manufactured products; however, prior studies suggest that majority of women use commercially manufactured douching products [30, 38]. Lastly, there may be exposure misclassification since we only had access to single, spot urine measurements and phthalates have a short half-life in the human body [3,4]. Previous studies have shown that women’s exposures to MEP and MnBP are relatively stable, with greater variability between individuals than within individuals [54]. However, use of feminine hygiene products is likely episodic, varying across the menstrual cycle. Unfortunately, NHANES only measures whether a product was or was not used in the last month, so we were not able to determine time since product use (or frequency of use) very precisely. This measurement error is likely nondifferential and would bias our results towards the null. Given the potential for nondifferential misclassification, the robust finding between vaginal douching and MEP levels is quite remarkable*.*

Our study also has several notable strengths. To our knowledge, this study is one of the few, if not only, to examine the association between self-reported use of feminine hygiene products and urinary biomarkers of chemical exposure. Our study population was large and included racial/ethnic, geographic, and socioeconomic diversity since it was derived from a nationally representative sample. This is also one of the first studies to identify potential mediating factors of racial/ethnic disparities in phthalates exposures using appropriate statistical techniques.

## Conclusions

Results of our study suggest that vaginal douching may be a source of human exposure to DEP among frequent users and contribute to racial/ethnic disparities in DEP exposure. This study adds weight to existing recommendations from health professionals that discourage the practice of douching. Future studies should confirm these findings in contemporary populations with more precise data and include assessment of other endocrine-disrupting chemicals that may be used in vaginal douches. Additionally, further research is needed on the adverse reproductive health consequences of chemical exposures originating from feminine hygiene products that are used in and around the vaginal area.

## Abbreviations

DEP: Diethyl phthalate
DnBP: Di-*n*-butyl phthalate
MEP: Monoethyl phthalate
MnBP: Mono-n-butyl phthalate
SES: Socioeconomic status
NHANES: National Health and Nutrition Examination Survey
CDC: US Centers for Disease Control and Prevention
LOD: Limit of detection
CI: Confidence interval
BMI: Body mass index
PIR: Household poverty-income ratio

## References

[1] Koniecki D, Wang R, Moody RP, Zhu J (2011). Phthalates in cosmetic and personal care products: Concentrations and possible dermal exposure. Environ Res.

[2] Hubinger JC, Havery DC (2006). Analysis of consumer cosmetic products for phthalate esters. J Cosmet Sci.

[3] Koch HM, Preuss R, Angerer JD (2006). Di (2‐ethylhexyl) phthalate (DEHP): Human metabolism and internal exposure–an update and latest results1. Int J Androl.

[4] Koch H, Christensen K, Harth V, Lorber M, Brüning T (2012). Di-n-butyl phthalate (DnBP) and diisobutyl phthalate (DiBP) metabolism in a human volunteer after single oral doses. Arch Toxicol.

[5] Woodruff TJ, Zota AR, Schwartz JM (2011). Environmental chemicals in pregnant women in the US: NHANES 2003–2004. Environ Health Perspect.

[6] Lee BM, Koo HJ (2007). Hershberger assay for antiandrogenic effects of phthalates. J Toxicol Environ Health.

[7] Hannas BR, Howdeshell KL, Furr J (2013). In utero phthalate effects in the female rat: A model for MRKH syndrome. Toxicol Lett.

[8] Kumar N, Sharan S, Srivastava S, Roy P (2014). Assessment of estrogenic potential of diethyl phthalate in female reproductive system involving both genomic and non-genomic actions. Reprod Toxicol.

[9] Sathyanarayana S, Barrett E, Butts S, Wang C, Swan SH (2014). Phthalate exposure and reproductive hormone concentrations in pregnancy. Reproduction.

[10] Braun JM, Just AC, Williams PL, Smith KW, Calafat AM, Hauser R. Personal care product use and urinary phthalate metabolite and paraben concentrations during pregnancy among women from a fertility clinic. J Expo Sci Environ Epidemiol. 2013;1–8. 10.1038/jes.2013.69 PMC4016195 24149971

[11] Swan SH, Main KM, Liu F, Stewart SL, Kruse RL, Calafat AM (2005). Decrease in anogenital distance among male infants with prenatal phthalate exposure. Environ Health Perspect.

[12] Swan SH, Liu F, Hines M, Kruse RL, Wang C, Redmon JB (2010). Prenatal phthalate exposure and reduced masculine play in boys. Int J Androl.

[13] James-Todd T, Stahlhut R, Meeker JD, Powell S, Hauser R, Huang T (2012). Urinary phthalate metabolite concentrations and diabetes among women in the national health and nutrition examination survey (NHANES) 2001–2008. Environ Health Perspect.

[14] Reddy BS, Rozati R, Reddy S, Kodampur S, Reddy P, Reddy R (2006). High plasma concentrations of polychlorinated biphenyls and phthalate esters in women with endometriosis: A prospective case control study. Fertil Steril.

[15] Guo Y, Weck J, Sundaram R, Goldstone AE, Buck Louis G, Kannan K (2014). Urinary concentrations of phthalates in couples planning pregnancy and its association with 8-hydroxy-2’-deoxyguanosine, a biomarker of oxidative stress: Longitudinal investigation of fertility and the environment study. Environ Sci Technol.

[16] Sprague BL, Trentham-Dietz A, Hedman CJ, Wang J, Hemming JD, Hampton JM (2013). Circulating serum xenoestrogens and mammographic breast density. Breast Cancer Res.

[17] Serrano SE, Karr CJ, Seixas NS, Nguyen RH, Barrett ES, Janssen S (2014). Dietary phthalate exposure in pregnant women and the impact of consumer practices. Int J Environ Res Public Health.

[18] Wolff MS, Teitelbaum SL, Pinney SM, Windham G, Liao L, Biro F (2010). Investigation of relationships between urinary biomarkers of phytoestrogens, phthalates, and phenols and pubertal stages in girls. Environ Health Perspect.

[19] Duty SM, Ackerman RM, Calafat AM, Hauser R (2005). Personal care product use predicts urinary concentrations of some phthalate monoesters. Environ Health Perspect.

[20] Silva MJ, Barr DB, Reidy JA, Malek NA, Hodge CC, Caudill SP (2004). Urinary levels of seven phthalate metabolites in the U.S. population from the national health and nutrition examination survey (NHANES) 1999–2000. Environ Health Perspect.

[21] Kobrosly RW, Parlett LE, Stahlhut RW, Barrett ES, Swan SH (2012). Socioeconomic factors and phthalate metabolite concentrations among united states women of reproductive age. Environ Res.

[22] Koo HJ, Lee BM (2004). Estimated exposure to phthalates in cosmetics and risk assessment. J Toxicol Environ Health.

[23] Morello-Frosch R, Zuk M, Jerrett M, Shamasunder B, Kyle AD (2011). Understanding the cumulative impacts of inequalities in environmental health: Implications for policy. Health Aff.

[24] Wormuth M, Scheringer M, Vollenweider M, Hungerbühler K (2006). What are the sources of exposure to eight frequently used phthalic acid esters in europeans?. Risk Anal.

[25] Parlett LE, Calafat AM, Swan SH (2013). Women’s exposure to phthalates in relation to use of personal care products. J Expo Sci Environ Epidemiol.

[26] Just AC, Adibi JJ, Rundle AG, Calafat AM, Camann DE, Hauser R (2010). Urinary and air phthalate concentrations and self-reported use of personal care products among minority pregnant women in new york city. J Expo Sci Environ Epidemiol.

[27] Romero-Franco M, Hernández-Ramírez RU, Calafat AM, Cebrián ME, Needham LL, Teitelbaum S (2011). Personal care product use and urinary levels of phthalate metabolites in mexican women. Environ Int.

[28] Buckley JP, Palmieri RT, Matuszewski JM, Herring AH, Baird DD, Hartmann KE (2012). Consumer product exposures associated with urinary phthalate levels in pregnant women. J Expo Sci Environ Epidemiol.

[29] Hines EP, Calafat AM, Silva MJ, Mendola P, Fenton SE (2009). Concentrations of phthalate metabolites in milk, urine, saliva, and serum of lactating north carolina women. Environ Health Perspect.

[30] Brotman RM, Klebanoff MA, Nansel T, Zhang J, Schwebke JR, Yu KF (2008). Why do women douche? A longitudinal study with two analytic approaches. Ann Epidemiol.

[31] Sarantis H, Naidenko O, Gray S, Houlihan J, Malkan S. Not so sexy: The health risks of secret chemicals in fragrance. Campaign for Safe Cosmetics. 2010. http://www.ewg.org/sites/default/files/report/SafeCosmetics_FragranceRpt.pdf Accessed 10 Oct 2014

[32] Houlihan J, Brody C, Schwan B. Not too pretty. Environmental Working Group. 2002. http://static.ewg.org/reports/2002/NotTooPretty.pdf?_ga=1.38970539.966727461.1421736393. Accessed 10 Oct 2014.

[33] US Centers for Disease Control and Prevention. National Health and Nutrition Examination Survey 2001–2002 Laboratory Procedure Manual: Phthalate Monoesters. http://www.cdc.gov/nchs/data/nhanes/nhanes_01_02/PHPYPA_b_met_phthalates.pdf. Accessed 2 April 2014.

[34] US Centers for Disease Control and Prevention. National Health and Nutrition Examination Survey 2003–2004 Laboratory Procedure Manual: Phthalate Metabolites. http://www.cdc.gov/nchs/data/nhanes/nhanes_03_04/l24ph_c_met.pdf. Accessed 2 April 2014.

[35] Barr DB, Wilder LC, Caudill SP, Gonzalez AJ, Needham LL, Pirkle JL (2005). Urinary creatinine concentrations in the US population: Implications for urinary biologic monitoring measurements. Environ Health Perspect.

[36] Iacobucci D (2012). Mediation analysis and categorical variables: The final frontier. J Consumer Psych.

[37] Baron RM, Kenny DA (1986). The moderator–mediator variable distinction in social psychological research: Conceptual, strategic, and statistical considerations. J Pers Soc Psychol.

[38] Grimley DM, Annang L, Foushee HR, Bruce FC, Kendrick JS (2006). Vaginal douches and other feminine hygiene products: Women’s practices and perceptions of product safety. Matern Child Health J.

[39] Ferranti M. An odor of racism: Vaginal deodorants in african-american beauty culture and advertising. Advertising & Society Review. 2011;11.

[40] Holzman C, Leventhal JM, Qiu H, Jones NM, Wang J (2001). Factors linked to bacterial vaginosis in nonpregnant women. Am J Public Health.

[41] Scholes D, Daling JR, Stergachis A, Weiss NS, Wang SP, Grayston JT (1993). Vaginal douching as a risk factor for acute pelvic inflammatory disease. Obstet Gynecol.

[42] Zhang J, Thomas AG, Leybovich E (1997). Vaginal douching and adverse health effects: A meta-analysis. Am J Public Health.

[43] American College of Obstetricians and Gynecologists (2006). Committee opinion no. 345: Vulvodynia. Obstet Gynecol.

[44] American College of Obstetricians and Gynecologists. Frequently asked questions FAQ077: Pelvic inflammatory disease. 2011. https://www.acog.org/-/media/For-Patients/faq077.pdf?dmc=1&ts=20150120T0233228590. Accessed 12 June 2014.

[45] American College of Obstetricians and Gynecologists (2013). Committee opinion no. 575: Exposure to toxic environmental agents. Obstet Gynecol.

[46] National Research Council (2008). Phthalates and Cumulative Risk Assessment: the Task Ahead.

[47] Engel SM, Miodovnik A, Canfield RL, Zhu C, Silva MJ, Calafat AM (2010). Prenatal phthalate exposure is associated with childhood behavior and executive functioning. Environ Health Perspect.

[48] Watkins DJ, Téllez-Rojo MM, Ferguson KK, Lee JM, Solano-Gonzalez M, Blank-Goldenberg C (2014). In utero and peripubertal exposure to phthalates and BPA in relation to female sexual maturation. Environ Res.

[49] Dodson RE, Nishioka M, Standley LJ, Perovich LJ, Brody JG, Rudel RA (2012). Endocrine disruptors and asthma-associated chemicals in consumer products. Environ Health Perspect.

[50] Zota A, Calafat A, Woodruff T (2014). Temporal trends in phthalate exposures: Findings from the national health and nutrition examination survey, 2001–2010. Environ Health Perspect.

[51] Scranton A. Chem fatale: Potential health effects of toxic chemicals in feminine care products. Women’s Voices for the Earth. 2013. http://www.womensvoices.org/wp-content/uploads/2013/11/Chem-Fatale-Report.pdf. Accessed 10 Oct 2014.

[52] Tourgeman DE, Gentzchein E, Stanczyk FZ, Paulson RJ (1999). Serum and tissue hormone levels of vaginally and orally administered estradiol. Obstet Gynecol.

[53] Onerdonk AB, Delaney ML, Hinkson PL, Dubois AM (1992). Quantitative and qualitative effects of douche preparations on vaginal microflora. Obstet Gynecol.

[54] Hoppin JA, Brock JW, Davis BJ, Baird DD (2002). Reproducibility of urinary phthalate metabolites in first morning urine samples. Environ Health Perspect.

